# Investigating the impact of physical activity on BMI, motor skills, and sleep patterns in elementary school children

**DOI:** 10.3389/fpubh.2025.1695317

**Published:** 2025-11-21

**Authors:** Xin Li, Bin Ma

**Affiliations:** College of Sports Science, Tianjin Normal University, Tianjin, China

**Keywords:** PA, body mass index, motor skills, sleep patterns, children

## Abstract

**Objective:**

This research examines how interschool physical activity (PA) interventions affect children’s BMI, motor skills, and sleep behaviors.

**Methods:**

In this investigation, a stratified random sampling method was implemented to choose 40 physically and mentally healthy fifth-grade students (average age of 10.72 ± 0.56 years) from an elementary school in Tianjin, China, after stratification by gender, randomly divided into experimental group (EG, *n* = 20, 10 boys and 10 girls) and control groups (CG, *n* = 20, 10 boys and 10 girls). The EG participated in 320 min of physical activity (PA) weekly, comprising 200 min of standard physical education classes and 120 min of supplementary intervention activities. The CG adhered to the standard timetable, consisting of 200 min of physical education and 120 min of non-PA weekly. BMI, motor skills (evaluated through standardized assessments like the 50 m run, 50 m × 8 back-and-forth run, 1-min jump rope, sit-and-reach and 1-min sit-ups), PA (moderate- to-vigorous-intensity PA (MVPA) and sedentary time), and sleep patterns (duration and efficiency) were measured at weeks 0, 6, and 12.

**Results:**

After the intervention, the BMI of the EG was significantly reduced by 0.26 kg/m^2^ (*p* < 0.05), all the indexes of motor skills were significantly improved (*p* < 0.05) compared to the CG. The EG significantly increased MVPA time by 30.28 min (*p* < 0.05), while significantly reducing sedentary time by 60.22 min (*p* < 0.01). Regarding sleep patterns, the EG slept an additional 44.83 min (*p* < 0.01), with sleep efficiency improving by 5.62% (*p* < 0.01). No significant variations were observed in any of these indicators within the CG. Concerning gender differences, boys and girls in the EG displayed similar trends in BMI, motor skills, PA levels, and sleep quality, with no notable differences.

**Conclusion:**

Enhancing interschool PA by 120 min weekly over a 12-week period has been shown to significantly improve BMI, motor skills, PA levels, and sleep quality among 10-to-11-year-old children. This intervention offers a practical foundation for promoting children’s health and developing school physical education standards.

## 1 Introduction

PA refers to any bodily movement produced by skeletal muscle contraction that requires energy expenditure (1). It involves engaging various parts of the body through movement and motion to maintain and promote physical health. PA plays a crucial role in children’s growth and development. It not only supports physical health but also fosters mental well-being and social interaction skills (1). Research indicates that PA further enhances children’s physical fitness by improving cardiorespiratory fitness and motor skills, factors essential for their long-term healthy development (2). The issue of childhood PA is a worldwide concern, as many nations are witnessing a decrease in activity levels (3). China is facing similar challenges with its youth experiencing reduced PA (4). Studies show that 80% of kids and teens globally do not achieve the advised 60 min of moderate PA each day. (5). China’s latest physical fitness survey report highlights that significant health concerns persist among students, including high rates of poor vision and myopia, rising occurrence of overweight and obesity, and declining grip strength levels (6). According to cross-sectional data from a study of 114,072 children aged 9–12 in China, 94.88% of the children are significantly less active on a daily basis (7). The elementary school years represent a critical period for children’s development and growth. This stage’s healthy development creates a robust foundation for future physical and mental wellness (8). Recognizing the need to encourage an active lifestyle from a young age, schools have implemented programs that effectively promote PA in children. By offering a wide range of physical activities in elementary schools, children can spend more time participating in sports, which helps them develop better overall physical health (8). A study using multivariate data analysis with 2,407 students (aged 11–17) in southeastern China explored the links between engaging in sports, exercises that enhance muscle strength, and active commuting with self-evaluated physical fitness, which demonstrated that enhanced physical fitness was related to more frequent sports participation and increased involvement in muscle-strengthening activities (9). These researches usually involve multiple components, targeting PA levels along with health-related elements like body mass index, motor skills and sleep patterns (10, 11).

PA significantly influences BMI regulation in children. A study of 396 children (214 girls and 182 boys) aged 7.6 years old in eastern Kansas revealed that children who engaged in MVPA through a greater number of short (5–10 min) and medium-to-long periods (≥10 min) were associated with lower BMI percentiles and waist circumferences compared to fewer periods (12). In another cross-sectional study of 220 children (91 boys and 129 girls) in the grades 5–6 of elementary school, a significant inverse linear trend was observed between body fat parameters and incremental PA levels, indicating that higher PA was associated with lower body fat (13). Moreover, the link between PA, BMI, and related metabolic factors differs between genders. In a study of 150 children who are 12 to 13 years old, including 69 boys and 81 girls, boys had significantly higher values in BMI, waist circumference, and levels of low, moderate, and vigorous PA compared to girls. Girls, on the other hand, had higher serum levels of triglycerides, insulin, and HOMA-IR, suggesting that exercise intervention is particularly important for girls to promote general health (14). Sedentary behavior also influence BMI (15). A study involving 264 children aged 7 to 10 found a positive link between television viewing duration and increased BMI evaluations and waist circumference, emphasizing the importance of limiting screen time to support a healthy BMI (15). While current research has made some progress in examining the impact of PA on BMI, it has primarily focused on PA in daily life. Few investigations have focused on the influence of school-based programs on children’s BMI regulation and this study aims to address this gap.

Motor skills development in elementary school children is significantly influenced by PA. A comprehensive review of 44 studies revealed robust evidence indicating a negative correlation between motor proficiency and body weight status, alongside a positive correlation between motor proficiency and cardiorespiratory and musculoskeletal fitness (16). Zheng et al. (17) used accelerometers to measure MVPA duration among 1,752 students aged 6–18 years and physical fitness was assessed according to the National Student Physical Fitness Standards (2014 Edition, China). The study found that increase of average daily MVPA duration was strongly linked to higher assessments of physical fitness. Similarly, a games-based PA professional learning program (PLUNGE) in primary schools improved basic movement abilities and PA during class in stage three students (18). These findings suggest that targeted PA interventions can enhance motor skills development in elementary school children, which in turn may have long-term benefits for their health and PA participation.

The correlation between PA and sleep behaviors among elementary school children is a burgeoning research focus. A study of 458 preschool children found that physical activity primarily acted as a protective factor for sleep duration, while sedentary behavior posed a risk to nocturnal sleeping period (19). A cross-sectional research conducted by Aoki et al. (20). involving 3,123 children (1,558 boys and 1,565 girls, aged 12.5 ± 1.2 years) revealed that for boys aged 10–12, participating in a minimum of 60 min of MVPA daily was inversely related to daytime sleepiness, and among 12–14-year-old girls, performing at least 60 min of MVPA daily was negatively correlated with inadequate weekend sleep duration. Various factors, in conjunction with PA, can influence sleep patterns. A cross-sectional analysis by Chaput et al. (21) included 5,777 children aged 9–11 from 12 regions found an inverse relationship between sleep duration and MVPA, sedentary behavior, and poor dietary habits; sleep efficiency displayed negative correlations with MVPA and unhealthy dietary patterns, and a positive correlation with sedentary time; and a delayed bedtime was positively associated with sedentary time, screen time, and unhealthy dietary patterns, while demonstrating negative associations with MVPA and a healthy dietary pattern. These observations pointed to the multifaceted connections between PA, sleep, and other lifestyle practices in children attending elementary school. Current research on the impact of PA on children’s sleep primarily consists of cross-sectional studies, while research on how school-based interventions affect children’s sleep regulation is relatively limited, and this study seeks to contribute to this area.

This study aims to evaluate the effectiveness of a school-based PA program in enhancing overall health by examining related health indicators. The research objectives focus on analyzing how the intervention impacts primary school children’s PA, BMI, motor skills, and sleep routines. The hypothesis proposed that increasing extracurricular activity classes to three 40-min sessions per week, totaling 320 min of physical education weekly over 12 weeks (22.23), H1: PA interventions in children significantly negatively impact BMI. H2: PA interventions in children significantly impact motor skills. H3: PA interventions in children significantly impact sleep behavior.

## 2 Methods

### 2.1 Participants

Using a stratified random sampling approach, 44 healthy fifth-grade children were selected from a primary school in Tianjin, China. After excluding one child with chronic conditions (cardiovascular disease, kidney disease, asthma, etc.), physical disabilities, or intellectual disabilities, informed consent documents were provided to 43 children and their guardians based on voluntary participation. The study’s objectives and procedures were explained, resulting in 40 children and their guardians agreeing to participate. Ultimately, 40 children (20 boys and 20 girls), aged 10 to 11 years with an average age of 10.72 ± 0.56, were included as valid samples. After stratification by gender, the participating children were randomly divided into an experimental group (EG, *n* = 20, 10 boys and 10 girls) and a control group (CG, *n* = 20, 10 boys and 10 girls). EG, Hight (1.53 ± 0.13) m, Weight (42.62 ± 3.87) kg, BMI (18.22 ± 1.96) kg/m^2^; CG, Hight (1.52 ± 0.12) m, Weight (42.07 ± 3. 41) kg, BMI (18.24 ± 1. 67) kg/m^2^. No significant difference was observed between the two groups prior to intervention. Initial sample size calculations were conducted using G*Power software version 3.1, with an effect size of 0.25, statistical power of 0.95, and a significance level of *p* < 0.05. At least 14 samples were required for each group, and the 20 samples in each group in this study met this requirement. A minimum of 14 participants per group was determined to be necessary, a criterion that was satisfied by having 20 participants per group in the study. The Ethics Committee of the College of Sports Science at Tianjin Normal University approved the research.

### 2.2 Study design

In this study, the EG engaged in a 12-week intervention comprising 320 min of PA weekly, consisting of 200 min of standard physical education and an additional 120 min of intervention (22, 23). The CG maintained their routine, involving 200 min per week, supplemented by 120 min of non-physical extracurricular activities such as language or art.

During the intervention phase for the EG, each 40-min PA session comprised three parts as outlined below (24):

1. First part (∼ 10 min): Warm-up activities. Participants engaged in activities such as jogging and radio gymnastics to thoroughly warm up, with flexibility exercises appropriately incorporated to help students adjust their heart rates, enter an active state, and prevent injuries (22, 24).
2. Second part (∼ 25 min): Main activities. The main activities comprised a 50 m run, 50 m × 8 back-and-forth run, a 1-min jump rope session, a 1-min sit-up exercise, basketball, soccer, and various sports games. Each session focused on one or several of these activities, conducted at a moderate to high exercise intensity level (22, 24).
3. Third Part (∼ 5 min): Cool-down Section. Engaging in relaxation activities and incorporating suitable stretching exercises can expedite muscle recovery and preserve muscle function (22, 24).

During the exercise intervention, to ensure their average heart rate was within the moderate intensity range, Polar heart rate monitors (Finland) were worn by school-aged children to observe their heart rates in real time (22, 23). The target heart rate (Target HR) for moderate intensity was calculated using the Karvonen formula based on maximum heart rate (HRmax) and resting heart rate (HR rest): Target HR = (HRmax − HR rest) × [60–69%] + HR rest, HRmax = 220 − age (25).

BMI, PA levels, motor skills, and sleep patterns were assessed in both the EG and CG at weeks 0, 6, and 12 of the investigation (26). Detailed information about the study was provided to participants and their parents or legal guardians, such as its aim, length, type of intervention, and any potential risks and benefits, before data was collected. Significantly, in the event of injuries or unfavorable occurrences, parents or legal guardians would receive prompt notification following the approved ethical guidelines, enabling them to seek medical attention from their family doctor or pediatrician. Participants were notified that they could withdraw from the study at any time without any adverse effects. Legal guardians gave written consent, and the children provided written assent.

### 2.3 Variables and measuring instruments

#### 2.3.1 Body mass index

Height was assessed with a stadiometer (TZG, Shanghai). Verify that the stadiometer is properly calibrated prior to commencing the test. The child stands upright on the pedals of the stadiometer with his/her back against the vertical backboard. The child’s hips, shoulder blades and head are in contact with the vertical backboard. The child’s head is kept in the Frankfurt level position. Measurement readings should be recorded to the nearest 0.1 cm. Measure weight using the electronic weight scale (FG2415LB). Calibrate the electronic weight scale before the test begins. The child stood at the center of the scale platform, and weight was recorded to the nearest 0.1 kg. BMI was calculated using the formula BMI = weight/height^2^ (kg/m^2^) (27).

#### 2.3.2 PA and sleep patterns

PA levels were measured with the triaxial accelerometer (ActiGraph GT3X) on three occasions: pre-intervention (PRE), during the intervention (DUR), and post-intervention (POST), with participants wearing the device for 7 consecutive days. The accelerometer was fixed to the child’s right anterior superior iliac spine and was only taken off for activities such as bathing, water-related events, and contact sports. For valid data analysis, a minimum of 4 days of recording is required, including three weekdays and one weekend day, with at least 8 h of data per day (28, 29). Wear-time validation followed Troiano’s default parameters (30, 31). Researchers conducted daily telephone follow-ups to determine whether participants were using the accelerometer as instructed and whether the wear time met the required duration. Accelerometer data were processed using specified cut-points to capture children’s spontaneous and intermittent activities accurately (32). Periods of 20 min with zero activity were classified as non-wear time, while counts below 0/min or above 15,000/min were excluded as biologically implausible (33). PA intensity levels (sedentary, light, moderate, vigorous) were determined based on child-specific thresholds proposed by Evenson et al. sedentary condition (≤100 counts/min), light activity (101–2,295 counts/min), and MVPA (≥2,296 counts/min) (34–36). Additionally, the triaxial accelerometers were utilized to evaluate sleep patterns, with participants wearing the device on their non-dominant wrist during nocturnal sleep (37–39). Throughout each assessment phase (PRE, DUR, POST), over a week, nightly recordings were made of sleep parameters, including how long participants slept and their sleep efficiency, which is the percentage of time asleep while in bed (40–42).

#### 2.3.3 Motor skills assessment

Test children’s physical fitness according to the assessment items and requirements outlined in “National Student Physical Fitness Standards (2014 Revision, China)” (43). The assessment items for fifth-grade elementary school students include BMI, 50 m run, 50 m × 8 back-and-forth run, 1-min jump rope, sit-and-reach and 1-min sit-ups, and sit-and-reach, reflecting students’ physical fitness indicators. Every test was quantitative, not significantly limited by age, and focused on the child’s attainable motor skills. Prior to the assessment, testing conditions were established, and children participated in all tests in groups of 10. Each participant went through a standardized 10-min warm-up routine before starting the tests (44). Before conducting the tests, examiners received training, with the following criteria being standardized: (1) Each test technique was demonstrated and explained verbally with proficiency. (2) Before the test was conducted, each participant went through each task. (3) The child was advised to perform the task to the utmost of their ability according to the instructions (e.g., “as fast as you can” for the 50-m run, “as far as possible” for the sit-and-reach) (45). During the testing process, this study assigned additional specialists and qualified experts to oversee the control of testing procedures. The equipment used for the test included a standard running track, a seated forward bending tester (TMQ, Beijing), a skipping rope (QSZW, Chengdu), a professional stopwatch (YS, Shenzhen). All assessments are quantitative and have no significant age-related developmental ceiling effect. The test results were corresponded to the scores of the National Physical Fitness Standards, and the average of the five scores was calculated as the exercise capacity (17).

### 2.4 Quality control

The study plan and execution have been thoroughly standardized to ensure high quality and accurate data. Teachers and referees involved in this project were systematically trained prior to the start of the survey to eliminate as many confounding variables as possible. To guarantee the accuracy of the data used for analysis, a strict data cleaning protocol was established for this project. In the course of data preprocessing, data were checked for logical errors, missing information, inaccuracies, or ambiguities and were retested or removed as needed to ensure the data set’s authenticity and validity. Before the start of the PA intervention, the teaching plan for the entire intervention period must be established and strictly followed to ensure the continuity and integrity of the curriculum. Coaches must not be replaced midway through the program. During instruction, coaches must control the pace and timing of teaching to ensure that each service component was scheduled appropriately, avoiding excessive delays or rushing through the program. Coaches must establish clear teaching objectives and steps, as well as corresponding teaching materials and equipment, and must not improvise during class. During testing, coaches and referees must comply with the testing schedule, strictly adhere to the testing schedule to participate in testing on time, be familiar with the correct testing methods, requirements, and standards for each project, strictly follow operating procedures during testing.

### 2.5 Statistical analyses

Data was analyzed utilizing SPSS 26.0 software. The Shapiro–Wilk test was applied to determine the distribution of samples for BMI, PA, exercise capacity, and sleep patterns in both the EG and CG at each evaluation stage (26). Line chart were employed to compare these variables between the two groups across the three time points (26). Independent samples t-tests were conducted to examine the differences in variables between groups at each time interval (24). Statistical significance was determined with a threshold of *p* < 0.05 for every analysis (25).

## 3 Results

Table 1 presents the participants’ height, weight, and BMI characteristics. For the PRE moment, the EG and CG did not differ significantly in terms of height, weight, and BMI (*p* > 0.05). In the POST test, the EG showed a significant increase in height by approximately 0.03 m and a decrease in BMI by about 0.26 kg/m^2^ compared to the PRE test (*p* < 0.05). For the POST moment, children in the EG had a BMI that was 0.31 kg/m^2^ lower than that in the CG (*p* < 0.05). There were no notable changes in the height, weight, and BMI of the CG between the PRE and POST tests (*p* > 0.05). A 12-week PA intervention might effectively reduce children’s BMI. Regarding gender differences, boys and girls in the EG exhibited similar trends in changes to height, weight, and BMI. Boys demonstrated greater magnitude of change at week 12, suggesting they might be more responsive to this intervention.

**Table 1:** Participants’ height, weight and BMI (*n* = 40).

Variable and category		PRE (0th week)		DUR (6th week)		POST (12th week)	
		CG	EG	CG	EG	CG	EG
Height (m), *M*(SD)	Group (*n* = 20)	1.52(0.06)	1.53(0.07)	1.53(0.06)	1.54(0.08)	1.54(0.04)	1.56(0.06)^a^
	Boy (*n* = 10)	1.53(0.05)	1.52(0.05)	1.54(0.05)	1.53(0.06)	1.55(0.03)	1.55(0.04)^a^
	Girl (*n* = 10)	1.51(0.04)	1.54(0.06)	1.52(0.04)	1.55(0.07)	1.53(0.03)	1.57(0.05)^a^
Weight (kg), *M*(SD)	Group (*n* = 20)	42.15(3.92)	42.65(4.14)	42.71(3.75)	43.02(4.82)	43.32(5.09)	43.70(3.84)
	Boy (*n* = 10)	43.69(3.07)	43.04(3.22)	44.29(2.98)	43.38(4.07)	44.91(4.54)	43.84(3.27)
	Girl (*n* = 10)	40.61(2.85)	42.26(3.01)	41.13(2.56)	42.66(3.59)	41.73(3.26)	43.56(2.93)
BMI (kg/m^2^), *M*(SD)	Group (*n* = 20)	18.24(1.69)	18.22(1.73)	18.25(1.81)	18.14(1.79)	18.27(1.62)	17.96(1.59)^ab^
	Boy (*n* = 10)	18.66(1.13)	18.63(1.24)	18.68(1.27)	18.53(1.21)	18.69(1.17)	18.25(1.05)^ab^
	Girl (*n* = 10)	17.81(1.06)	17.82(1.18)	17.80(1.02)	17.76(1.07)	17.83(1.12)	17.67(0.98)^ab^

Significantly differences compared to EG in PRE. ^a^*p* < 0.05. Significantly differences compared to CG in POST. ^b^*p* < 0.05.

Table 2 presents the motor skills traits of the individuals involved. In the PRE test, the EG and CG exhibited no significant differences in motor skills assessments, such as the 50 m run, 50 m × 8 back-and-forth run, 1-min jump rope, sit-and-reach, 1-min sit-ups, and overall motor skills scores.

**Table 2:** Participants’ motor skills (*n* = 40).

Variable and category		PRE (0th week)		DUR (6th week)		POST (12th week)	
		CG	EG	CG	EG	CG	EG
50 m run (s), M(SD)	Group (*n* = 20)	9.58(0.68)	9.62(0.73)	9.52(0.75)	9.46(0.67)	9.41(0.81)	9.10(0.69)^ac^
	Boy (*n* = 10)	9.37(0.47)	9.43(0.56)	9.31(0.51)	9.24(0.53)	9.20(0.57)	8.83(0.55)^ac^
	Girl (*n* = 10)	9.79(0.59)	9.81(0.62)	9.73(0.60)	9.68(0.61)	9.62(0.64)	9.37(0.54)^ac^
50 × 8 back-and-forth run (s), M(SD)	Group (*n* = 20)	114.27(8.82)	114.47(9.38)	113.49(9.95)	112.56(9.64)^a^	112.82(9.12)	109.76(9.03)^bd^
	Boy (*n* = 10)	111.23(7.53)	111.37(8.06)	110.45(8.47)	109.29(8.19)^a^	109.74(8.24)	106.43(7.81)^bd^
	Girl (*n* = 10)	117.31(6.97)	117.57(7.75)	116.53(7.51)	115.83(7.63)^a^	115.90(7.87)	113.09(7.32)^bd^
1-min jump rope (reps), M(SD)	Group (*n* = 20)	113.59(8.26)	112.36(8.07)	115.24(8.56)	118.93(9.86)^a^	117.62(9.26)	126.51(9.58)^bd^
	Boy (*n* = 10)	112.63(6.83)	111.85(6.52)	114.32(6.92)	118.02(7.26)^a^	116.57(7.65)	126.79(7.72)^bd^
	Girl (*n* = 10)	114.55(6.95)	112.87(6.79)	116.16(7.84)	119.84(7.39)^a^	118.67(7.96)	126.23(7.36)^bd^
Sit-and-reach (cm), M(SD)	Group (*n* = 20)	12.84(1.37)	12.63(1.31)	12.94(1.46)	13.06(1.43)	13.05(1.58)	13.97(1.53)^ac^
	Boy (*n* = 10)	10.67(1.14)	10.45(1.05)	10.74(1.20)	10.82(1.16)	10.79(1.25)	11.76(1.23)^ac^
	Girl (*n* = 10)	15.01(1.21)	14.81(1.19)	15.14(1.32)	15.30(1.26)	15.31(1.33)	16.18(1.36)^ac^
1-min sit-ups (reps), M(SD)	Group (*n* = 20)	36.07(3.56)	36.32(3.34)	36.58(3.95)	37.25(3.86)	37.16(3.77)	38.94(3.69)^ac^
	Boy (*n* = 10)	35.84(2.41)	36.01(2.53)	36.29(2.62)	36.83(2.93)	36.72(2.84)	38.37(2.75)^ac^
	Girl (*n* = 10)	36.30(2.26)	36.63(2.73)	36.87(2.46)	37.67(2.58)	37.60(2.99)	39.51(2.63)^ac^
Motor skills score (points), M(SD)	Group (*n* = 20)	79.38(7.49)	77.79(7.81)	78.85(7.57)	79.68(7.65)	79.46(7.34)	82.78(6.82)^ac^
	Boy (*n* = 10)	79.85(5.95)	78.46(5.83)	79.38(5.64)	80.34(5.19)	79.93(5.28)	83.42(5.07)^ac^
	Girl (*n* = 10)	78.91(5.46)	77.12(5.52)	78.32(5.15)	79.02(5.60)	78.99(5.39)	82.14(4.75)^ac^

Significantly differences compared to EG in PRE. ^a^*p* < 0.05; ^b^*p* < 0.01. Significantly differences compared to CG in POST. ^c^*p* < 0.05; ^d^*p* < 0.01.

During the DUR and POST test phases, the EG demonstrated significant improvements compared to the PRE phase, completing the 50 × 8 back-and-forth run faster by approximately 1.91 s (*p* < 0.05) during DUR and 4.71 s (*p* < 0.01) during POST. Additionally, they performed more repetitions in the 1-min jump rope test, with increases of approximately 6.57 reps (*p* < 0.05) during DUR and 14.15 reps (*p* < 0.01) during POST. Comparing PRE and POST test moments, the EG reduced 0.52 s in 50 m run, increased 2.62 reps in 1-min sit-ups, extended 1.34 cm in sit-and-reach, with notable differences (*p* < 0.05) (Table 2).

Table 2 demonstrated that during the POST test phase, the EG outperformed the CG in multiple motor skills assessments. Children in the EG showed improved performance compared to the CG, with 0.31 s faster in the 50-meter run, 3.06 s quicker in the 50 × 8 back-and-forth run, 8.89 reps more in the 1-min jump rope, 1.78 reps additional in the 1-min sit-ups, 0.92 cm greater distance in the sit-and-reach test, and 3.32 points higher in motor skill scores. The differences were significant according to statistical analysis (*p* < 0.05).

Regarding the CG, no notable differences were observed in any of the tests between PRE and POST for the children (*p* > 0.05) (Table 2).

Following a 12-week physical activity intervention, the EG demonstrated a 4.99-point increase in motor skill scores, suggesting a potentially significant impact on motor skills.

With respect to gender differences, both boys and girls in the EG showed similar patterns in their performance changes in the 50 m run, 50 m × 8 back-and-forth run, 1-min jump rope, sit-and-reach, 1-min sit-ups, and overall motor skills scores, with no notable differences. At weeks 6 and 12, boys showed a larger degree of change, indicating they might respond better to this intervention.

Table 3 shows the trends in changes in MVPA, sedentary time, sleep duration and efficiency between the EG and CG during the PRE, DUR, and POST test phases. In the PRE test moment, EG and CG had no significant differences in these indicators (*p* > 0.05).

**Table 3:** Participants’ MVPA, sedentary and sleep patterns (*n* = 40).

Variable and category		PRE (0th week)		DUR (6th week)		POST (12th week)	
		CG	EG	CG	EG	CG	EG
MVPA (min/d), M(SD)	Group (*n* = 20)	47.20(4.67)	44.69(4.36)	54.87(4.98)	70.18(6.82)^bd^	49.21(4.74)	74.97(7.25)^bf^
	Boy (*n* = 10)	49.85(3.34)	46.91(3.27)	56.57(3.63)	71.64(5.15)^bd^	51.79(3.52)	76.93(5.41)^bf^
	Girl (*n* = 10)	44.55(3.06)	42.47(2.99)	53.17(3.42)	68.72(4.73)^bd^	46.63(3.19)	73.01(4.88)^bf^
Sedentary (min/d), M(SD)	Group (*n* = 20)	378.97(32.71)	382.48(35.49)	384.16(36.64)	332.13(31.05)^bd^	373.24(34.76)	322.26(31.87)^bf^
	Boy (*n* = 10)	372.52(23.29)	378.39(24.63)	378.47(25.09)	328.62(23.47)^bd^	368.73(23.85)	318.96(22.16)^bf^
	Girl (*n* = 10)	385.42(22.37)	386.57(24.96)	389.85(26.15)	335.64(22.38)^bd^	377.75(24.02)	325.56(23.29)^bf^
Sleep duration (min/d), M(SD)	Group (*n* = 20)	528.96(51.33)	525.12(50.89)	526.82(50.46)	567.05(54.72)^bd^	534.13(55.26)	569.95(57.08)^bf^
	Boy (*n* = 10)	532.57(34.18)	528.39(33.62)	529.46(35.01)	569.24(36.07)^bd^	533.74(35.31)	570.61(34.78)^bf^
	Girl (*n* = 10)	525.35(32.67)	521.85(32.73)	524.18(33.85)	564.86(33.56)^bd^	534.52(34.14)	569.29(34.22)^bf^
Sleep efficiency (%), M(SD)	Group (*n* = 20)	89.34(9.21)	87.43(8.92)	90.52(9.13)	92.85(10.46)^ac^	90.16(9.37)	93.05(9.49)^ae^
	Boy (*n* = 10)	89.76(6.86)	87.81(6.74)	90.75(6.52)	93.04(7.37)^ac^	90.58(6.41)	93.17(6.91)^ae^
	Girl (*n* = 10)	88.92(6.50)	87.05(5.89)	90.29(6.98)	92.66(7.12)^ac^	89.74(6.26)	92.93(6.65)^ae^

Significantly differences compared to EG in PRE. ^a^*p* < 0.05; ^b^*p* < 0.01. Significantly differences compared to CG in DUR. ^c^*p* < 0.05; ^d^*p* < 0.01. Significantly differences compared to CG in POST. ^e^*p* < 0.05; ^f^*p* < 0.01.

The research reveals that, relative to the CG, the EG involved in significantly more MVPA by 15.31 min during the DUR phase and 25.76 min during the POST phase (*p* < 0.01). Additionally, they dedicated much less time to sedentary behaviors, with reductions of about 52.03 min during the DUR phase and 50.98 min during the POST phase (*p* < 0.01) per day. Regarding sleep patterns, children from the EG demonstrated an increase in nightly sleep duration by 40.23 min and 35.82 min (*p* < 0.01) during the DUR and POST phases respectively, in contrast to the CG, along with enhancements in sleep efficiency by 2.33 and 2.89% (*p* < 0.05) (Table 3).

Contrasting the PRE test, the EG increased by 25.49 min and 30.28 min in MVPA time per day during the DUR and POST (*p* < 0.01), and spent 50.35 min and 60.22 min less in sedentary time per day during the DUR and POST (*p* < 0.01). Concerning sleep, the EG exhibited a significant increase in sleep duration of 41.93 min and 44.83 min per night during the DUR and POST compared to the PRE, respectively (*p* < 0.01), with an improvement in sleep efficiency of 5.42 and 5.62% (*p* < 0.01) (Table 3).

For CG, compared with PRE, the amount of time spent on MVPA per day increased by 2.01 min in the POST stage, while sedentary time decreased by 5.73 min, the amount of sleep per night increased by 5.17 min, and sleep efficiency decreased by 0.82%, with no significant differences (*p* > 0.05) (Table 3).

The 12-week intervention in PA may increase daily MVPA and nightly sleep duration, potentially yielding significant impacts on children’s health outcomes.

Concerning gender differences, boys and girls in the EG had similar trends in MVPA, sedentary time, sleep duration, and sleep efficiency changes, with boys demonstrating a greater change at weeks 6 and 12, indicating they might be more receptive to the intervention.

## 4 Discussion

This research aimed to assess how a physical activity program within school affects children’s height, weight, BMI, motor skills, physical activity levels, and sleep patterns. The PA intervention led to a substantial positive impact on children’s physical health, as demonstrated by comparisons between the EG and CG during the intervention. The study results demonstrate that the EG achieved statistically significant improvements in BMI and motor skills, along with positive changes in physical activity levels and sleep patterns. The enhancements made the EG more consistent with the World Health Organization’s standards for children aged 10–11, illustrating the specific health benefits of PA interventions on their overall health. Schools should ensure every student has access to sufficient PA to maintain a healthy BMI, enhance motor skills, and improve sleep patterns. Education authorities and school administrators should prioritize the comprehensiveness and diversity of physical education curricula, offering activities tailored to different age groups and physical abilities. Additionally, schools should establish dedicated assessment mechanisms to regularly monitor students’ physical condition and participation in PA, enabling timely adjustments and optimization of PE programs. Furthermore, collaborating with families and communities to foster a supportive environment for PA will help increase student participation and cultivate lifelong exercise habits.

### 4.1 PA and BMI in elementary school children

PA is crucial in managing children’s BMI. An analysis of data from two provincial surveys of fifth-grade children in Canada demonstrated a negative link between BMI and PA levels (46). A study involving 318 s-and-third-grade students in USA, revealed that children participating in MVPA with a greater proportion of sessions lasting 10 to less than 15 and 15 min or more exhibited lower BMI percentiles and waist circumferences compared to peers with fewer such sessions (47). Another study with a cross-sectional design, involving 150 elementary school students aged 3–5 years, showed a significant negative linear trend between body fat parameters and increasing levels of physical activity, implying that increased PA is associated with decreased body fat (48). This study’s outcomes were consistent with previous findings, revealing that the EG spent an extra 30.28 min per day on MVPA and reduced sedentary time by 60.22 min compared to the PRE, leading to a height gain of 0.03 m and a reduction in BMI by 0.26 kg/m^2^. The findings suggest that consistent monitoring and intervention in children’s PA levels can contribute to sustaining a healthy BMI. There is an association between PA, BMI, and related metabolic factors. Conducted over 3 years, the PA in the Curriculum (PAAC) study was a cluster randomized controlled trial involving 24 elementary schools, all second- and third-grade students followed through fourth and fifth grades. The research was introduced a 90-min weekly program integrating MVPA into the educational program and revealed a notably lower increase in BMI over 3 years in schools where PAAC was conducted for ≥75 min per week compared to schools with less than 75 min per week. This finding indicates the potential effectiveness of this strategy in mitigating BMI increase over a three-year period (49).

The mechanisms by which PA influences BMI involve complex processes encompassing neuromodulation, physiological adaptation, and metabolic regulation (50–52). In terms of neuromodulation, PA regulates appetite by affecting the central nervous system, thereby controlling energy intake and expenditure (50). High-intensity interval training enhances neuroplasticity by raising the levels of brain-derived neurotrophic factor and enhancing neural activity in the ventromedial hypothalamic nucleus. This neuroplasticity correlates with enhanced fat oxidation and visceral lipolysis (51). Through physiological adaptation, PA influences hepatic gluconeogenesis by modulating the TRB3/Akt interaction in insulin-resistant states. This process occurs by inhibiting TRB3 protein activity, thereby reducing hepatic glycogenesis and maintaining glucose homeostasis (53). PA stimulates heat production in fat tissue and influences hormonal pathways, boosting the ability of major metabolic organs like skeletal muscle and liver to burn fat and sustain energy equilibrium (50). Regarding metabolic regulation, PA reduces body fat and BMI by increasing energy expenditure and improving insulin sensitivity. PA improves hypothalamic inflammatory states by reducing obesity-associated pro-inflammatory mediators (e.g., free fatty acids, TNFα, adiponectin, and advanced glycation end-products), thereby restoring leptin sensitivity and decreasing food intake (52). PA reverses obesity-related brain structural abnormalities by inducing metabolic and neurotrophic plasticity changes in brain structures, ultimately enhancing metabolic health (54). PA influences BMI through multiple mechanisms including neuromodulation, physiological adaptation, and metabolic regulation. The synergistic effects of these mechanisms not only aid BMI management but also positively impact overall metabolic health. Childhood represents a critical stage for physical development, and moderate PA can boost basal metabolism, enhance muscle mass, and aid in maintaining a healthy weight. The generalizability of this study’s findings is evident across different age groups of children and diverse cultural backgrounds, where similar trends are observed. Therefore, school policymakers should consider increasing the duration and intensity of weekly physical education classes to ensure all students have opportunities to engage in physical exercise, particularly in light of the growing prevalence of childhood obesity.

### 4.2 PA and motor skills development in elementary school children

PA is essential for the development of motor skills among children. A review of 40 studies found significant positive correlations between motor skills and both cardiorespiratory and musculoskeletal fitness, alongside significant negative correlations with weight status (55). Similarly, the implementation of the game-based PA, conducted with third grade students, improved participants basic motor skills and increased levels of PA within the classroom (56). These studies support the outcomes of our research. Following 12 weeks of PA, the EG showed marked improvements in the 50 m run, 50 m × 8 back-and-forth run, 1-min jump rope, sit-and-reach, and 1-min sit-ups tests relative to the PRE test (*p* < 0.01). The research suggests that interventions centered on PA can successfully enhance motor skills in children, potentially benefiting their health and PA involvement over time. Research on children aged 5–6 showed that the Walk, an eight-month-long PA curriculum facilitated by classroom and physical education instructors, led to substantial improvements in motor competence and PA levels (57). A study by Bi et al. (58) demonstrated that among 184 randomly selected adolescents aged 12.87 years (84 males, 100 females) from a school, engaging in 40-min sessions of moderate-to-high-intensity physical education classes three times weekly for 12 weeks produced a meaningful increase in maximal oxygen uptake and effective improvement in cardiorespiratory endurance. The process of developing motor skills through PA involves a coordinated mechanism between the nervous and muscular systems: the nervous system promotes motor skill development by regulating muscle activity, while the muscular system supports movement through coordinated muscle actions and force output (59). The advancement of physical skills requires the active participation of myelin formation within the central nervous system. Oligodendrocytes, continuously generated within the brain, form myelin sheaths (60). These cells, along with the myelin they generate play a crucial role in motor skills learning and development (60). The nervous system generates specific motor behaviors by activating muscle synergist groups (4). The modular organization of these synergist groups forms the foundation of motor actions (61). This structure enables the nervous system to produce diverse movements through flexible combinations of synergist groups and to optimize motor skill development by reorganizing synergist groups (62). Motor skill development also involves information processing within the sensory system, which aids in adjusting and refining motor skills through feedback mechanisms (63). The synergistic interaction of these mechanisms enables physical activities to promote the development and enhancement of motor skills. A deeper understanding of these mechanisms facilitates the development of more effective interventions to foster motor skill development in elementary school students.

### 4.3 PA and sleep patterns in elementary school children

The effects of PA on sleep remain a subject of considerable debate in current research (64–66). Our study observed that during the POST test, children from the EG exhibited a 67.76% increase in MVPA, a 8.54% increase in sleep duration, and a 5.62% enhancement in sleep efficiency relative to the PRE. There was a clear correlation between PA and sleep quality, as MVPA levels were linked to improved sleep quality and longer sleep duration. Multiple studies align with the findings of this research. Ezati et al. (67) recruited 67 female college students aged 18–26 to participate in 8 weeks of moderate-to-high-intensity aerobic exercise, performed three times weekly for 60 min per session. The study revealed that 8 weeks of moderate-to-high-intensity exercise significantly improved both sleep duration and sleep quality. Junior et al. (68) recruited 23 males aged 15–17 years to perform moderate-intensity functional training twice weekly over a 6-week intervention period. Results indicated that 6 weeks of functional training significantly improved sleep quality in these males. A study by Chaput et al. (21) involving 5,777 children aged 9–11 revealed a negative link between sleep duration and sedentary time, showing that later bedtimes were related to more sedentary activities and less MVPA. PA can influence sleep patterns in elementary school children through various biological mechanisms. In terms of neural regulation, PA promotes the secretion of dopamine, serotonin, melatonin, and other neurotransmitters, regulates body temperature, improves circadian rhythms, and enhances sleep quality (69). Regarding psychological regulation, PA reduces depressive moods, alleviates academic stress, and lowers the incidence of sleep disorders (70). In physiological regulation, the rise and subsequent drop in body temperature after exercise facilitates sleep onset during the cooling phase. Regular PA improves cardiac function, enhancing blood oxygen-carrying capacity and aiding nighttime relaxation and recovery. Furthermore, exercise-induced muscle fatigue increases deep sleep duration, which is crucial for bodily restoration (71, 72). Regarding metabolic adaptation, PA elevates basal metabolic rate, enabling the body to expend more energy at night and potentially shortening wakefulness periods within the sleep cycle. Simultaneously, PA improves insulin sensitivity and lowers blood glucose levels, which are necessary for maintaining stable energy levels and good sleep quality (73). PA influences sleep patterns through multiple mechanisms—neurological, physiological, and metabolic adaptations—which collectively promote better sleep quality and overall health. Encouraging moderate PA serves as a powerful approach to enhance sleep quality and advance health (71, 72).

## 5 Strengths and limitations

This study evaluates the influence of interschool PA strategies employed on children’s BMI, motor skills, PA levels, and sleep patterns, offering theoretical and practical insights for enhancing children’s physical fitness and health. This research was carefully planned, utilizing a randomized controlled trial to minimize confounding factors and confirm the intervention’s impact by comparing the EG and CG. The assessment indexes of this study were comprehensive, with simultaneous monitoring of BMI, motor skills, PA level and sleep pattern, revealing the overall impact of PA on multiple aspects of children’s health. The intervention program of this study was practically feasible, based on the design of school scenarios, the combination of extra activities and regular physical education classes, easy to operate and easy to promote, and provided a model that could be used for physical education interventions in primary schools.

This research has certain limitations. The sample size was only 40 participants, which is relatively small and may inadequately portray the diversity of the intended population. This limitation restricts the generalizability of the results. Future studies should aim to increase the sample size to improve the study’s applicability and precision. During data collection, seasonal factors should be fully controlled to eliminate the impact of seasonal variations on physical activity levels. Potential selection bias should be considered in subject selection, addressing underlying biases such as health consciousness or lifestyle choices. Given that the elementary school semester spans approximately 16 weeks, the 12-week intervention period in this study was insufficient to assess long-term effects. The absence of long-term data may lead to overestimation or underestimation of intervention effects, as certain long-term outcomes may not manifest within a short timeframe. Additionally, adherence variations across different contexts (e.g., work, family, leisure) require consideration. Contextual factors like weather, social support, and academic stress significantly influence physical activity behaviors, warranting in-depth analysis in future studies. The generalizability of findings requires validation through cross-cultural and cross-regional research. It is imperative to investigate the consistency of exercise intervention effects across different geographical and cultural contexts, as well as identify any specific factors that may require adjustment.

## 6 Conclusion

This study reveals the multidimensional positive effects of a PA on the health of 10–11 years old children, including a significant reduction in BMI, improved motor skills, increased PA levels, and enhanced sleep patterns. This 12-week intervention program, featuring 320 min of weekly physical activity (combining regular physical education classes and supplemental activities), not only substantially elevated children’s overall health status but also established a robust foundation for their healthy development and well-being. These findings hold profound implications for educational policy and public health guidelines, underscore the importance of integrating PA within school settings and provide robust evidence supporting strategies to increase both the duration and intensity of PA to promote children health. The study’s results call on policymakers to develop PA as a core component of school curricula and encourage public health guidelines to adopt evidence-based PA interventions. This creates an educational environment conducive to children’s health, offering a replicable and scalable model for global child health promotion initiatives that advance worldwide child health standards.

## Data Availability

The original contributions presented in the study are included in the article/supplementary material, further inquiries can be directed to the corresponding author.

## References

[1] Bingham DD Morris JL Lewis K Foweather L Goss H O'Brien W . Children's perceptions of physical literacy: exploring meaning, value, and capabilities for lifelong physical activity. Front Sports Act Living. (2025) 7:1548546. doi: 10.3389/fspor.2025.1548546 40438339 PMC12116515

[2] Ré AHN Cattuzzo MT Stodden D Santos GD Tertuliano ALO Monteiro CBM . Physical development and sociocultural context influences on children’s physical activity. Rev Paul Pediatr. (2025) 43:e2024113. doi: 10.1590/1984-0462/2025/43/2024113, PMID: 39841744 PMC11748495

[3] Brandão T Brites R Hipólito J Nunes O. Emotion goals, emotion regulation, and mental health: a mediational hypothesis. Clin Psychol. (2023) 27:290–301. doi: 10.1080/13284207.2023.2214312

[4] Guo Q Yao SY Wang XZ Wu YY. An experimental study on the influence of KDL curriculum on pupils' physical education learning interest and physical health level. J Phys Educ. (2025) 32:132–8. doi: 10.16237/j.cnki.cn44-1404/g8.2025.02.005

[5] Guthold R Stevens GA Riley LM Bull FC. Global trends in insufficient physical activity among adolescents: a pooled analysis of 298 population-based surveys with 1·6 million participants. Lancet Child Adolesc Health. (2020) 4:23–35. doi: 10.1016/S2352-4642(19)30323-2, PMID: 31761562 PMC6919336

[6] Department of Physical Health and Arts Education,Ministry of Education. Release report of the eighth national survey on student physical fitness and health. Chin J Sch Health. (2021) 42:1281–2. doi: 10.16835/j.cnki.1000-9817.2021.09.001

[7] Chen ST Liu Y Tremblay MS Hong JT Tang Y Cao ZB . Meeting 24-h movement guidelines: prevalence, correlates, and the relationships with overweight and obesity among Chinese children and adolescents. J Sport Health Sci. (2021) 10:349–59. doi: 10.1016/j.jshs.2020.07.002, PMID: 32679341 PMC8167320

[8] Wang XZ Yang YG Kong L Tong TT Chen MY. A study on the development strategy for children and adolescents’ sports health promotion in China. J Chengdu Sport Univ. (2020) 46:6–12. doi: 10.15942/j.jcsu.2020.03.002

[9] Shi C Chen S Wang L Yan J Liang K Hong J . Associations of sport participation, muscle-strengthening exercise and active commuting with self-reported physical fitness in school-aged children. Front Public Health. (2022) 10:873141. doi: 10.3389/fpubh.2022.873141, PMID: 35937209 PMC9355271

[10] Liang G Wang LJ Chen H Lin H Chen Y. The association of the body mass index of children with 24-hour activity composition and isotemporal substitution: a compositional data analysis. China Sport Sci. (2022) 42:77–84. doi: 10.16469/j.css.202203008

[11] Fairclough SJ Hurter L Dumuid D Gába A Rowlands AV Cruz BDP . The physical behaviour intensity Spectrum and body mass index in school-aged youth: a compositional analysis of pooled individual participant data. Int J Environ Res Public Health. (2022) 19:8778. doi: 10.3390/ijerph19148778 35886629 PMC9320124

[12] Willis EA Ptomey LT Szabo-Reed AN Honas JJ Lee J Washburn RA . Length of moderate-to-vigorous physical activity bouts and cardio-metabolic risk factors in elementary school children. Prev Med. (2015) 73:76–80. doi: 10.1016/j.ypmed.2015.01.022, PMID: 25647532 PMC4455886

[13] Hong HR Ha CD Jin YY Kang HS. The effect of physical activity on serum IL-6 and vaspin levels in late elementary school children. J Exerc Nutr Biochem. (2015) 19:99–106. doi: 10.5717/jenb.2015.15060507, PMID: 26244128 PMC4523811

[14] Hong HR Ha CD Kong JY Lee SH Song MG Kang HS. Roles of physical activity and cardiorespiratory fitness on sex difference in insulin resistance in late elementary years. J Exerc Nutr Biochem. (2014) 18:361–9. doi: 10.5717/jenb.2014.18.4.361, PMID: 25671203 PMC4322027

[15] Robinson S Daly RM Ridgers ND Salmon J. Screen-based behaviors of children and cardiovascular risk factors. J Pediatr. (2015) 167:1239–45. doi: 10.1016/j.jpeds.2015.08.067, PMID: 26434372

[16] Cattuzzo MT Dos Santos Henrique R Ré AH de Oliveira IS Melo BM de Sousa Moura M . Motor competence and health related physical fitness in youth: a systematic review. J Sci Med Sport. (2014) 19:123–9. doi: 10.1016/j.jsams.2014.12.004 25554655

[17] Zheng N, Wang LJ, Zheng DH, Chen H, Liang G, Qiu YP. Relationship between weekly physical activity patterns and physical fitness among children and adolescents in China. J Shanghai Uni Sport (2025). 49:91–102. doi:doi: 10.16099/j.sus.2024.08.01.0002.

[18] Miller A Christensen EM Eather N Sproule J Annis-Brown L Lubans DR. The PLUNGE randomized controlled trial: evaluation of a games-based physical activity professional learning program in primary school physical education. Prev Med. (2015) 74:1–8. doi: 10.1016/j.ypmed.2015.02.002, PMID: 25668220

[19] Chang Z Lei W. A study on the relationship between physical activity, sedentary behavior, and sleep duration in preschool children. Front Public Health. (2021) 9:618962. doi: 10.3389/fpubh.2021.618962, PMID: 33898373 PMC8059703

[20] Aoki T Fukuda K Tanaka C Kamikawa Y Tsuji N Kasanami R . The relationship between sleep habits, lifestyle factors, and achieving guideline-recommended physical activity levels in ten-to-fourteen-year-old Japanese children: a cross-sectional study. PLoS One. (2020) 15:e0242517. doi: 10.1371/journal.pone.0242517, PMID: 33186410 PMC7665581

[21] Chaput JP Katzmarzyk PT LeBlanc AG Tremblay MS Barreira TV Broyles ST . Associations between sleep patterns and lifestyle behaviors in children: an international comparison. Int J Obes Suppl. (2015) 5:S59–65. doi: 10.1038/ijosup.2015.21, PMID: 27152187 PMC4850622

[22] Yan J Qian KJ Tao BL Zhang WJ Zhong BB Jiang YY. An experimental study on the effects of exercise intervention on mental health of children: the chain-mediating role of physical self-esteem and self-concept. Sports Sci. (2022) 43:89–96. doi: 10.13598/j.issn1004-4590.2022.03.010

[23] Duan K Liu Y Zhang D Zhang YD Sun JT Zhang Y. Effects of 12 weeks of on-line aerobic combined resistance exercise on physiologic function and motor quality in children with simple obesity. Chin J Clin Pharmacol. (2023) 39:3262–5. doi: 10.13699/j.cnki.1001-6821.2023.22.015

[24] Li S Chen C. Effects of an exercise intervention on executive function and academic performance of school-aged children. J Mud Nor Uni. (2023) 3:64–8. doi: 10.13815/j.cnki.jmtc(ns).2023.03.005

[25] Yan Y. Analysis of exercise density and sports load testing in primary school physical education classes - taking Jiuting no.4 primary School in Songjiang District, Shanghai as an example. New Educ. (2024). S2:184–185. doi:CNKI:SUN:LXJY.0.2024-S2-088.

[26] Costa JA Vale S Cordovil R Rodrigues LP Cardoso V Proença R . A school-based physical activity intervention in primary school: effects on physical activity, sleep, aerobic fitness, and motor competence. Front Public Health. (2024) 12:1365782. doi: 10.3389/fpubh.2024.1365782, PMID: 38444436 PMC10912631

[27] Hui L Xinnan Z. Growth standardized values and curves based on weight for length/height, body mass index for Chinese children under 7 years of age. Chin J Pediatr. (2009) 47:281–5. doi: 10.3760/cma.j.issn.0578-1310.2009.04.011 19555567

[28] Migueles JH Cadenas-Sanchez C Ekelund U Delisle Nystrom C Mora-Gonzalez J Lof M . Accelerometer data collection and processing criteria to assess physical activity and other outcomes: a systematic review and practical considerations. Sports Med. (2017) 47:1821–45. doi: 10.1007/s40279-017-0716-0 28303543 PMC6231536

[29] Evenson KR Catellier DJ Gill K Ondrak KS Mcmurray RG. Calibration of two objective measures of physical activity for children. J Sports Sci. (2008) 26:1557–65. doi: 10.1080/02640410802334196, PMID: 18949660

[30] Troiano RP. Large-scale applications of accelerometers: new frontiers and new questions. Med Sci Sports Exerc. (2007) 39:1501. doi: 10.1097/mss.0b013e318150d42e 17805080

[31] Kuritz A Mall C Schnitzius M Mess F. Physical activity and sedentary behavior of children in afterschool programs: an accelerometer-based analysis in full-day and half-day elementary schools in Germany. Front Public Health. (2020) 8:463. doi: 10.3389/fpubh.2020.00463 32984249 PMC7492590

[32] Cliff DP Reilly JJ Okely AD. Methodological considerations in using accelerometers to assess habitual physical activity in children aged 0-5 years. J Sci Med Sport. (2009) 12:557–67. doi: 10.1016/j.jsams.2008.10.008, PMID: 19147404

[33] Esliger D Copeland J Barnes J Tremblay MS. Standardizing and optimizing the use of accelerometerdata for free-living physical activity monitoring. J Phys Act Health. (2005) 2:366–83. doi: 10.1123/jpah.2.3.366

[34] Steene-Johannessen J Hansen BH Dalene KE Kolle E Northstone K Møller NC . Variations in accelerometry measured physical activity and sedentary time across Europe - harmonized analyses of 47,497 children and adolescents. Int J Behav Nutr Phys Act. (2020) 17:38. doi: 10.1186/s12966-020-00930-x, PMID: 32183834 PMC7079516

[35] Cooper AR Goodman A Page AS Sherar LB Esliger DW Van Sluijs EM . Objectively measured physical activity and sedentary time in youth: the international children's accelerometry database (ICAD). Int J Behav Nutr Phys Act. (2015) 12:113. doi: 10.1186/s12966-015-0274-5 26377803 PMC4574095

[36] Trost SG Loprinzi PD Moore R Pfeiffer KA. Comparison of accelerometer cut points for predicting activity intensity in youth. Med Sci Sports Exerc. (2011) 43:1360–8. doi: 10.1249/MSS.0b013e318206476e 21131873

[37] Meltzer LJ Walsh CM Traylor J Westin AM. Direct comparison of two new actigraphs and polysomnography in children and adolescents. Sleep. (2012) 35:159–66. doi: 10.5665/sleep.1608 22215930 PMC3242684

[38] Alsaadi SM Mcauley JH Hush JM Bartlett DJ Mckeough ZM Grunstein RR . Assessing sleep disturbance in low back pain: the validity of portable instruments. PLoS One. (2014) 9:e95824. doi: 10.1371/journal.pone.0095824 24763506 PMC3998977

[39] Smith C Galland B Taylor R Meredith-Jones K. ActiGraph GT3X+ and Actical wrist and hip worn accelerometers for sleep and wake indices in young children using an automated algorithm: validation with polysomnography. Front Psych. (2019) 10:958. doi: 10.3389/fpsyt.2019.00958 PMC6970953 31992999

[40] Ohayon M Wickwire EM Hirshkowitz M Albert SM Avidan A Daly FJ . National Sleep Foundation's sleep quality recommendations: first report. Sleep Health. (2017) 3:6–19. doi: 10.1016/j.sleh.2016.11.006 28346153

[41] Sadeh A Sharkey KM Carskadon MA. Activity-based sleep-wake identification: an empirical test of methodological issues. Sleep. (1994) 17:201–7. doi: 10.1093/sleep/17.3.201 7939118

[42] Hirshkowitz M Whiton K Albert SM Alessi C Bruni O DonCarlos L . National Sleep Foundation's sleep time duration recommendations: methodology and results summary. Sleep Health. (2015) 1:40–3. doi: 10.1016/j.sleh.2014.12.010 29073412

[43] Ministry of Education of China. Notice of the Ministry of Education on issuing 《National Student Physical Fitness Standards (2014 revision)》. (2024). Available online at: http://www.moe.gov.cn/s78/A17/twys_left/moe_938/moe_792/s3273/201407/t20140708_171692.html

[44] Rodrigues LP Luz C Cordovil R Bezerra P Silva B Camoes M . Normative values of the motor competence assessment (MCA) from 3 to 23 years of age. J Sci Med Sport. (2019) 22:1038–43. doi: 10.1016/j.jsams.2019.05.009, PMID: 31151877

[45] Rodrigues LP Stodden DF Lopes VP. Developmental pathways of change in fitness and motor competence are related to overweight and obesity status at the end of primary school. J Sci Med Sport. (2016) 19:87–92. doi: 10.1016/j.jsams.2015.01.002, PMID: 25660571

[46] Colapinto CK Rossiter M Khan MK Kirk SF Veugelers PJ. Obesity, lifestyle and socio-economic determinants of vitamin D intake: a population-based study of Canadian children. Can J Public Health. (2014) 105:e418–24. doi: 10.17269/cjph.105.4608, PMID: 25560887 PMC6972174

[47] Ward S Chow AF Humbert ML Bélanger M Muhajarine N Vatanparast H . Promoting physical activity, healthy eating and gross motor skills development among preschoolers attending childcare centers: process evaluation of the healthy start-Départ santé intervention using the RE-AIM framework. Eval Program Plann. (2018) 68:90–8. doi: 10.1016/j.evalprogplan.2018.02.005, PMID: 29505965

[48] Okely AD Kariippanon KE Guan H Taylor EK Suesse T Cross PL . Global effect of COVID-19 pandemic on physical activity, sedentary behavior and sleep among 3- to 5-year-old children: a longitudinal study of 14 countries. BMC Public Health. (2021) 21:940. doi: 10.1186/s12889-021-10852-3, PMID: 34001086 PMC8128084

[49] Donnelly JE Greene JL Gibson CA Smith BK Washburn RA Sullivan DK . Physical activity across the curriculum (PAAC): a randomized controlled trial to promote physical activity and diminish overweight and obesity in elementary school children. Prev Med. (2009) 49:336–41. doi: 10.1016/j.ypmed.2009.07.022, PMID: 19665037 PMC2766439

[50] Zhang Y Wang R Liu T Wang R. Exercise as a therapeutic strategy for obesity: central and peripheral mechanisms. Meta. (2024) 14:589. doi: 10.3390/metabo14110589, PMID: 39590824 PMC11596326

[51] Cheng B Du J Tian S Zhang Z Chen W Liu Y. High-intensity interval training or lactate administration combined with aerobic training enhances visceral fat loss while promoting VMH neuroplasticity in female rats. Lipids Health Dis. (2024) 23:405. doi: 10.1186/s12944-024-02397-2, PMID: 39696579 PMC11653782

[52] Della Guardia L Codella R. Exercise restores hypothalamic health in obesity by reshaping the inflammatory network. Antioxidants. (2023) 12:297. doi: 10.3390/antiox12020297, PMID: 36829858 PMC9951965

[53] Marinho R Mekary RA Muñoz VR Gomes RJ Pauli JR de Moura LP. Regulation of hepatic TRB3/Akt interaction induced by physical exercise and its effect on the hepatic glucose production in an insulin resistance state. Diabetol Metab Syndr. (2015) 7:67. doi: 10.1186/s13098-015-0064-x, PMID: 26288661 PMC4539706

[54] Mueller K Möller HE Horstmann A Busse F Lepsien J Blüher M . Physical exercise in overweight to obese individuals induces metabolic- and neurotrophic-related structural brain plasticity. Front Hum Neurosci. (2015) 9:372. doi: 10.3389/fnhum.2015.00372, PMID: 26190989 PMC4486867

[55] Hung LS Tidwell DK Hall ME Lee ML Briley CA Hunt BP. A meta-analysis of school-based obesity prevention programs demonstrates limited efficacy of decreasing childhood obesity. Nutr Res. (2015) 35:229–40. doi: 10.1016/j.nutres.2015.01.002, PMID: 25656407

[56] Cesa CC Barbiero SM Petkowicz RO Martins CC Marques R Andreolla AA . Effectiveness of physical exercise to reduce cardiovascular risk factors in youths: a randomized clinical trial. J Clin Med Res. (2015) 7:348–55. doi: 10.14740/jocmr1700w, PMID: 25780484 PMC4356096

[57] Aivazidis D Venetsanou F Aggeloussis N Gourgoulis V Kambas A. Enhancing motor competence and physical activity in kindergarten. J Phys Act Health. (2019) 16:184–90. doi: 10.1123/jpah.2018-0260, PMID: 30696335

[58] Bi CJ Yin XJ Shi LJ Wu HP Wang JX Shan Y . Intervention effects of moderate and high intensities of classroom physical activity on cardiorespiratory fitness and executive function among junior grade one students in Tibetan. Chi J Sch Hea. (2024) 45:322–5. doi: 10.16835/j.cnki.1000-9817.2024083

[59] Cirillo J. Physical activity, motor performance and skill learning: a focus on primary motor cortex in healthy aging. Exp Brain Res. (2021) 239:3431–8. doi: 10.1007/s00221-021-06218-1, PMID: 34499187

[60] McKenzie IA Ohayon D Li H de Faria JP Emery B Tohyama K . Motor skill learning requires active central myelination. Science. (2014) 346:318–22. doi: 10.1126/science.1254960, PMID: 25324381 PMC6324726

[61] Zhao K Zhang Z Wen H Wang Z Wu J. Modular Organization of Muscle Synergies to achieve movement behaviors. J Healthc Eng. (2019) 2019:1–9. doi: 10.1155/2019/8130297, PMID: 31827741 PMC6885185

[62] Torricelli D De Marchis C d'Avella A Tobaruela DN Barroso FO Pons JL. Reorganization of muscle coordination underlying motor learning in cycling tasks. Front Bioeng Biotechnol. (2020) 8:800. doi: 10.3389/fbioe.2020.00800, PMID: 32760711 PMC7373728

[63] Mongold SJ Georgiev C Legrand T Bourguignon M. Afferents to action: cortical proprioceptive processing assessed with corticokinematic coherence specifically relates to gross motor skills. eNeuro. (2024) 11:ENEURO.0384-23.2023. doi: 10.1523/ENEURO.0384-23.2023, PMID: 38164580 PMC10849019

[64] Zhao H Lu C Yi C. Physical activity and sleep quality Association in Different Populations: a Meta-analysis. Int J Environ Res Public Health. (2023) 20:1864. doi: 10.3390/ijerph20031864, PMID: 36767229 PMC9914680

[65] Sejbuk M Mirończuk-Chodakowska I Witkowska AM. Sleep quality: a narrative review on nutrition, stimulants, and physical activity as important factors. Nutrients. (2022) 14:1912. doi: 10.3390/nu14091912, PMID: 35565879 PMC9103473

[66] Alnawwar MA Alraddadi MI Algethmi RA Salem GA Salem MA Alharbi AA. The effect of physical activity on sleep quality and sleep disorder: a systematic review. Cureus. (2023) 15:e43595. doi: 10.7759/cureus.43595, PMID: 37719583 PMC10503965

[67] Ezati M Keshavarz M Barandouzi ZA Montazeri A. The effect of regular aerobic exercise on sleep quality and fatigue among female student dormitory residents. BMC Sports Sci Med Rehabil. (2020) 12:44. doi: 10.1186/s13102-020-00190-z, PMID: 32774864 PMC7405354

[68] Junior IDS Nunes RSM de Luca Corrêa H Vieira E. Functional training program: the impact on depression, anxiety and sleep quality in adolescents. Sport Sci Health. (2020) 3:233–42. doi: 10.1007/s11332-020-00679-7

[69] Escames G Ozturk G Baño-Otálora B Pozo MJ Madrid JA Reiter RJ Exercise and melatonin in humans: reciprocal benefits. J Pineal Res (2011). 52(1):1–11. doi: 10.1111/j.1600-079x.2011.00924.x. 21848991

[70] Xu L Yan W Hua G He Z Wu C Hao M. Effects of physical activity on sleep quality among university students: chain mediation between rumination and depression levels. BMC Psychiatry. (2025) 25:7. doi: 10.1186/s12888-024-06450-3 39748322 PMC11697847

[71] Rai A Aldabbas M Veqar Z. Effect of physical activity on sleep problems in sedentary adults: a scoping systematic review. Sleep Biol Rhythms. (2023) 22:13–31. doi: 10.1007/s41105-023-00494-w, PMID: 38476845 PMC10899995

[72] Byun H Hwang S Yi E Rokni L. Understanding the relationship between sleep quality and physical activity: implications for healthy aging. Iran J Public Health. (2024) 53:2491–9. doi: 10.18502/ijph.v53i11.16952, PMID: 39619912 PMC11607159

[73] Anwardeen NR Naja K Almuraikhy S Sellami M Al-Amri HS Philip N . The influence of circadian rhythm disruption during Ramadan on metabolic responses to physical activity: a pilot study. Front Neurosci. (2025) 19:1542016. doi: 10.3389/fnins.2025.1542016, PMID: 40066156 PMC11891360

